# DECtp: Calling Differential Gene Expression Between Cancer and Normal Samples by Integrating Tumor Purity Information

**DOI:** 10.3389/fgene.2018.00321

**Published:** 2018-08-28

**Authors:** Weiwei Zhang, Haixia Long, Binsheng He, Jialiang Yang

**Affiliations:** ^1^School of Science, East China University of Technology, Nanchang, China; ^2^Department of Information Science and Technology, Hainan Normal University, Haikou, China; ^3^The First Affiliated Hosptial, Changsha Medical University, Changsha, China; ^4^College of Information Engineering, Changsha Medical University, Changsha, China; ^5^Icahn Institute for Genomics and Multiscale Biology, Icahn School of Medicine at Mount Sinai, New York, NY, United States

**Keywords:** differentially expressed genes, tumor purity, generalized least square, the Wald test, generalized least square

## Abstract

Identifying differentially expressed genes (DEGs) between tumor and normal samples is critical for studying tumorigenesis, and has been routinely applied to identify diagnostic, prognostic, and therapeutic biomarkers for many cancers. It is well-known that solid tumor tissue samples obtained from clinical settings are always mixtures of cancer and normal cells. However, the tumor purity information is more or less ignored in traditional differential expression analyses, which might decrease the power of differential gene identification or even bias the results. In this paper, we have developed a novel differential gene calling method called DECtp by integrating tumor purity information into a generalized least square procedure, followed by the Wald test. We compared DECtp with popular methods like *t*-test and limma on nine simulation datasets with different sample sizes and noise levels. DECtp achieved the highest area under curves (AUCs) for all the comparisons, suggesting that cancer purity information is critical for DEG calling between tumor and normal samples. In addition, we applied DECtp into cancer and normal samples of 14 tumor types collected from The Cancer Genome Atlas (TCGA) and compared the DEGs with those called by limma. As a result, DECtp achieved more sensitive, consistent, and biologically meaningful results and identified a few novel DEGs for further experimental validation.

## Introduction

Nowadays, RNA sequencing (RNA-Seq) has become a routine for measuring RNA expression levels (Mortazavi et al., 2008; Wang et al., 2009). Due to continuous improvements on sequencing accuracy and reduction on costs, this technology has revolutionized most fields in life sciences especially clinical medicine (Berger et al., 2010). Among many goals of RNA-Seq study, identifying differentially expressed genes (DEGs) between usually two conditions is probably the most common (Ritchie et al., 2015). Generally speaking, DEG analysis performs statistical analysis to discover significant gene expression changes between the experimental and control groups, which are critical for explaining transcriptomic changes incurred by experimental conditions. For instance, DEGs between normal and tumor samples help to study tumorigenesis, and have been routinely applied to identify diagnostic, prognostic, and therapeutic biomarkers for many cancers (Wu et al., 2013).

Over the past years, a number of statistical methods and softwares have been developed for identifying DEGs by considering the distributions of gene transcript abundance measured by read counts, Fragments Per Kilobase of transcript per Million (FPKM) (Trapnell et al., 2012), RNA-Seq by Expectation Maximization (RSEM) (Li and Dewey, 2011), and so on. Gene read counts usually follow a multinomial distribution, which can be approximated by a Poisson distribution, if they are independently sampled from a population with fixed fractions of genes. Consequently, the Poisson distribution has been widely assumed to test for differential expressions (Marioni et al., 2008; Wang et al., 2010). However, there is only one single parameter in the Poisson distribution, so the resulting statistical test does not control for the type-I error (Robinson and Smyth, 2007). To solve this so-called over-dispersion problem, the negative binomial (NB) distribution has been proposed to model count data (Anders and Huber, 2010; Zhou et al., 2011; McCarthy et al., 2012; Wu et al., 2013). Alternatively, the read counts can be converted to log2 transformed counts per million, for which the Bayes moderated Student's *t*-test and linear modeling methods like limma can be used. For instance, limma used a linear model to assess differential expression from microarray or RNA-Seq technologies by using multifactor designed experiments. It has a few advantages include stable on even small sample sizes and good in complex experiments with a variety of experimental conditions and predictors (Ritchie et al., 2015).

Differential expression analyses have been widely performed in cancer (Liang and Pardee, 2003). It is known that clinical tumor samples contain not only tumor cells but also tumor-associated normal epithelial and stromal cells, immune cells, and vascular cells (Joyce and Pollard, 2009), which play important roles in tumor growth, disease progression, and drug resistance (Hanahan and Weinberg, 2011; Junttila and de Sauvage, 2013). As a result, tumor purity, i.e., the percentages of cancer cells in solid tumor samples, is critical in genomic, transcriptomic, and methylation analyses in cancer (Aran et al., 2015; Zheng et al., 2017). For example, we recently developed InfiniumPurify by integrating tumor purity into differential methylation (DM) analysis, which significantly improved the accuracy of the DM identification (Zheng et al., 2017). In addition, we developed a rigorous statistical method InfiniumClust to perform sample clustering on DNA methylation data using tumor purity, which also exhibited superior accuracy (Zhang et al., 2017). There are also a few attempts to account for tumor purity in differential expression analysis (Wang et al., 2015; Shen et al., 2016) by adding it as an additive or semi-additive covariate in linear models (Aran et al., 2015). For example, contamDE proposed a few statistical models to call differential genes between unmatched or matched normal and tumor samples, in which the mean expression for a “contaminated” tumor cell sample follows a semi-additive pattern (Shen et al., 2016). Briefly, let *w*_*i*_ be the proportion of tumor cells in the ith tumor sample. For the jth gene, contamDE models the distribution of reads from normal cell samples as *N*_*ij*_~*NB*(*k*_*i*_μ_*j*_, ϕ_*j*_) and those from “contaminated” tumor samples as $T_{ij}\sim NB(k_{i}^{\prime }(\mu_{j}+w_{i}\delta_{j}),\phi_{j}),$ where NB denotes the negative binomial distribution, *k*_*i*_ and $k_{i}^{{}^{\prime }}$ are normalization size factors for normal and tumor samples, μ_*j*_ and μ_*j*_+*w*_*i*_δ_*j*_ are the adjusted means for normal and tumor samples, and ϕ_*j*_ is the dispersion. The DE is obtained by testing if δ_*j*_ is 0. UNDO is designed for deconvoluting array-based gene expression data of tumor samples (Wang et al., 2015), which models the mixing proportion of pure tumor and stroma cells as latent variables. However, tumor purity has multiplicative effects on gene expression, which might not be additive (Zheng et al., 2017). Thus, it is inadequate to simply treat tumor purity as an additive or semi-additive covariate in computational models.

To solve this problem, we have developed a novel method called *D*ifferential *E*xpression *C*aller by combining *t*umor *p*urity information (DECtp) to identify DEGs between tumor and normal samples. DECtp models expression profiles of tumor samples as a mixed Gaussian distribution, where the mixing proportion is tumor purity. With known or estimated tumor purity, differential expressions are then called based on a generalized least square procedure followed by the Wald test. We performed analyses on extensive simulated data with different sample sizes and noise levels and TCGA data of various cancers. DECtp achieves more accurate, consistent, and biologically meaningful results than those from other state of the art methods, such as limma (Ritchie et al., 2015).

## Materials and methods

Supposing that the input data consists of expression profiles of *N* genes on *n*_0_ normal and *n*_1_ cancer samples, we first transform the expression values on each sample group (by log2 transformation, quantile normalization, and so on) such that they will follow a Gaussian distribution. This transformation allows for the introduction of a linear model with Gaussian noise in subsequent steps.

Specifically, for any gene *i*, let *X*_*i*_ be its transformed expressions on all normal samples. We assume that $X_{i}\sim N\left(m_{i},\sigma_{i}^{2}\right)$, where *m*_*i*_ and $\sigma_{i}^{2}\;$represent the mean and variance of *X*_*i*_. Similarly, let *Y*_*i*_ be the transformed expressions on “pure” cancer samples for gene *i*, which also admits a normal distribution. Without loss of generality, we assume *Y*_*i*_ = *X*_*i*_+δ_*i*_, where δ_*i*_ represents the difference between cancer and normal samples. Clearly, δ_*i*_ is a random variable following a normal distribution with mean μ_*i*_ and variance $\tau_{i}^{2}$, i.e., $\delta_{i}\sim N\left(\mu_{i},\tau_{i}^{2}\right)$. Thus, differential genes could be inferred by the hypothesis test: *H*_0_:μ_*i*_ = 0. However in practice, the expression profile of “pure” cancer sample *Y*_*i*_ is not observed. Instead, the observed expressions of solid tumor samples are always a mixture of expressions on cancer and normal cells.

Let $Y_{i}^{{}^{\prime }}$ be the expression profile of gene *i* on observed tumor samples. For a tumor sample with known purity λ_*s*_ estimated by existing methods, we use $Y_{is}^{{}^{\prime }}$ to denote the expression of gene *i* on sample *s*. Then $Y_{is}^{{}^{\prime }}\;$can be modeled by a linear formula: $Y_{is}^{{}^{\prime }}=\left(1-\lambda_{s}\right)X_{is}+\lambda_{s}Y_{is}=\left(1-\lambda_{s}\right)X_{is}+\lambda_{s}\left(X_{is}+\delta_{is}\right)=X_{is}+\lambda_{s}\delta_{is}$, so $Y_{is}^{\prime }\sim N(m_{i}+\lambda_{s}\mu_{i},\sigma_{i}^{2}+\lambda_{s}^{2}\tau_{i}^{2})$. Clearly, the gene expression variance of tumor samples are greater than or equal to that of normal samples since $\sigma_{i}^{2}+\lambda_{s}^{2}\tau_{i}^{2}\geq \sigma_{i}^{2}$, and bias can arise when directly testing the mean difference between *X*_*is*_ and $Y_{is}^{{}^{\prime }}$ due to the influence of tumor purity. It is worth noting that tumor purity has multiplicative (instead of additive) effect (Zheng et al., 2017) on differential expression under this assumption. So previous DEG calling method modeling tumor purity as an additive covariate might be inappropriate (Aran et al., 2015).

To solve this problem, we propose a simple linear model and a generalized least square procedure by taking *X*_*is*_ and $Y_{is}^{{}^{\prime }}$ as input data. Specifically for gene *i*, the linear regression model is trained as follows: *Z*_*i*_ = *Wβ*_*i*_+ϵ_*i*_, where

$$Z_{i}=\left[\begin{array}{c} X_{i1}\\ X_{i2}\\ \vdots \\ X_{in_{0}}\\ {Y^{\prime }}_{i1}\\ {Y^{\prime }}_{i2}\\ \vdots \\ {Y^{\prime }}_{in_{1}} \end{array}\right],W=\left[\begin{array}{c} 1\\ 1\\ \vdots \\ 1\\ 1\\ 1\\ \vdots \\ 1 \end{array}\;\begin{array}{c} 0\\ 0\\ \vdots \\ 0\\ \lambda_{1}\\ \lambda_{2}\\ \vdots \\ \lambda_{n_{1}} \end{array}\right],\beta_{i}=\left[\begin{array}{c} m_{i}\\ \mu_{i} \end{array}\right],and\;\epsilon_{i}=\left[\begin{array}{c} \epsilon_{1}\\ \epsilon_{2}\\ \vdots \\ \epsilon_{n_{0}}\\ \epsilon_{n_{0}+1}\\ \epsilon_{n_{0}+2}\\ \vdots \\ \epsilon_{n_{0}+n_{1}} \end{array}\right].$$

Here, the (*n*_0_+*n*_1_) × 1 vector *Z*_*i*_ represents expressions from normal and tumor samples with the first *n*_0_ entries from normal samples, and the last *n*_1_ entries from tumor samples. In addition, *W* is a matrix of dimensionality (*n*_0_+*n*_1_) × 2 with the first column consisting of all 1 s and the second column consisting of *n*_0_ 0 s and *n*_1_ tumor purities (i.e., λ_1_, λ_2_, …, λ_*n*_1__) for respective tumor samples. β_*i*_ is the linear model parameter to be determined, and ϵ_*i*_ is the random error. The objective is to test *H*_0_:μ_*i*_ = 0.

The parameters can be fitted by a least square procedure to minimize $|Z_{i}-\left(W\beta_{i}+\epsilon_{i}\right){|}_{2}^{2}$. As a result, ${\hat{\beta }}_{i}={\left(W^{T}W\right)}^{-1}W^{T}Z_{i}≜HZ_{i}$ where *H* = (*W*^*T*^*W*)−1*W*^*T*^, and $var\left(\;{\hat{\beta }}_{i}\right)=Hvar\;\left(Z_{i}\right)H^{T}$. The variance of *Z*_*i*_ is $\left[\begin{array}{cc} \Sigma & 0\\ 0 & \Sigma^{\prime } \end{array}\right]$, where$\Sigma^{\prime }={\left[\begin{array}{ccc} \sigma^{2} & 0 & 0\\ 0 & \ddots & 0\\ 0 & 0 & \sigma^{2} \end{array}\right]}_{n_{1}\times n_{1}}$ and $\Sigma^{\prime }={\left[\begin{array}{ccc} {\sigma^{\prime }}^{2} & 0 & 0\\ 0 & \ddots & 0\\ 0 & 0 & {\sigma^{\prime }}^{2} \end{array}\right]}_{n_{1}\times n_{1}}$ So, $var(\;{\hat{\beta }}_{i})=Hvar(Z_{i})H^{T}=[H_{1}\;H_{2}]\left[\begin{array}{cc} \Sigma & 0\\ 0 & \Sigma^{\prime } \end{array}\right]\left[\begin{array}{c} H_{1}^{T}\\ H_{2}^{T} \end{array}\right]=H_{1}\Sigma H_{1}^{T}+H_{2}\Sigma^{\prime }H_{2}^{T}$, then $var\left({\hat{\beta }}_{i}\right)$ can be obtained with σ^2^ and σ′2, the residual variances from normal and cancer groups respectively. Given estimated ${\hat{\beta }}_{i}$, regression residuals are now $\hat{\epsilon }=Z_{i}-W{\hat{\beta }}_{i}$, and the residual variances from normal and cancer groups are obtained as $\sigma^{2}=\frac{\sum_{i=1}^{n_{0}}{\hat{\epsilon }}_{i}^{2}}{n_{0}-2}$, $\sigma^{{}^{\prime }2}=\frac{\sum_{i=n_{0}+1}^{n_{0}+n_{1}}{\hat{\epsilon }}_{i}^{2}}{n_{1}-2}$. We apply a shrinkage estimator similar to Cui et al. (2005) on the estimated cancer/normal variances, and obtained ${\tilde{\sigma }}^{2}\;$ and ${\tilde{\sigma }}^{{}^{\prime }2}$. The procedure shrinks all residual variances to the genometeric mean and stabilizes the estimates. After getting ${\hat{\beta }}_{i}$ and $var\left(\;{\hat{\beta }}_{i}\right)$, the Wald test statistics for testing *H*_0_:μ_*i*_ = 0 is calculated as $t_{i}=\frac{{\hat{\beta }}_{i_{\left[2\right]}}}{\sqrt{{var\left({\hat{\beta }}_{i}\right)}_{\left[2,2\right]}}}$, where ${\hat{\beta }}_{i_{\left[2\right]}}\;$is the second item of ${\hat{\beta }}_{i}$ and $\sqrt{{var\left({\hat{\beta }}_{i}\right)}_{\left[2,2\right]}}\;$is the element of the matrix $\sqrt{var\left({\hat{\beta }}_{i}\right)}$ at indices [2,2]. Finally, we assume the Wald test follow a *t* distribution with *n*_0_+*n*_1_−2 degrees of freedom, and the *p*-values can be obtained accordingly. False discovery rate (FDR) can be estimated using established procedures such as the Benjamini-Hochberg method (Benjamini et al., 2001).

## Results

We applied and compared DECtp with canonical DEG calling algorithms like limma on a few simulated datasets and cancer datasets downloaded from The Cancer Genome Atlas (https://cancergenome.nih.gov/). Before stepping into detailed analyses, it is insightful to first examine the relationship between gene expression and tumor purity.

### Correlation between gene expression and tumor purity

Through extensive analyses of the TCGA data, we discovered that the expression levels of many genes have strong correlation with tumor purity in cancer and the correlation increases with the difference of gene expressions between cancer and normal samples. Specifically, the tumor purities were downloaded from https://zenodo.org/record/253193, which were calculated by InfiniumPurify (Zhang et al., 2015; Zheng et al., 2017). InfiniumPurify for purity estimation is based on an important observation from the Illumina Infinium 450 k methylation data: the number of probes with intermediate methylation level is significantly greater in tumor samples than that in normal samples. InfiniumPurify first identifies a number of informative differentially methylated CpG sites (iDMCs) from cancer-normal comparison by using a non-parametric Wilcoxon Rank-Sum test and ANOVA analysis for each probe, and then estimates purity from the probability density of methylation levels of iDMCs.

#### Expression levels of many genes have strong correlation with tumor purity

**Figure 1:**
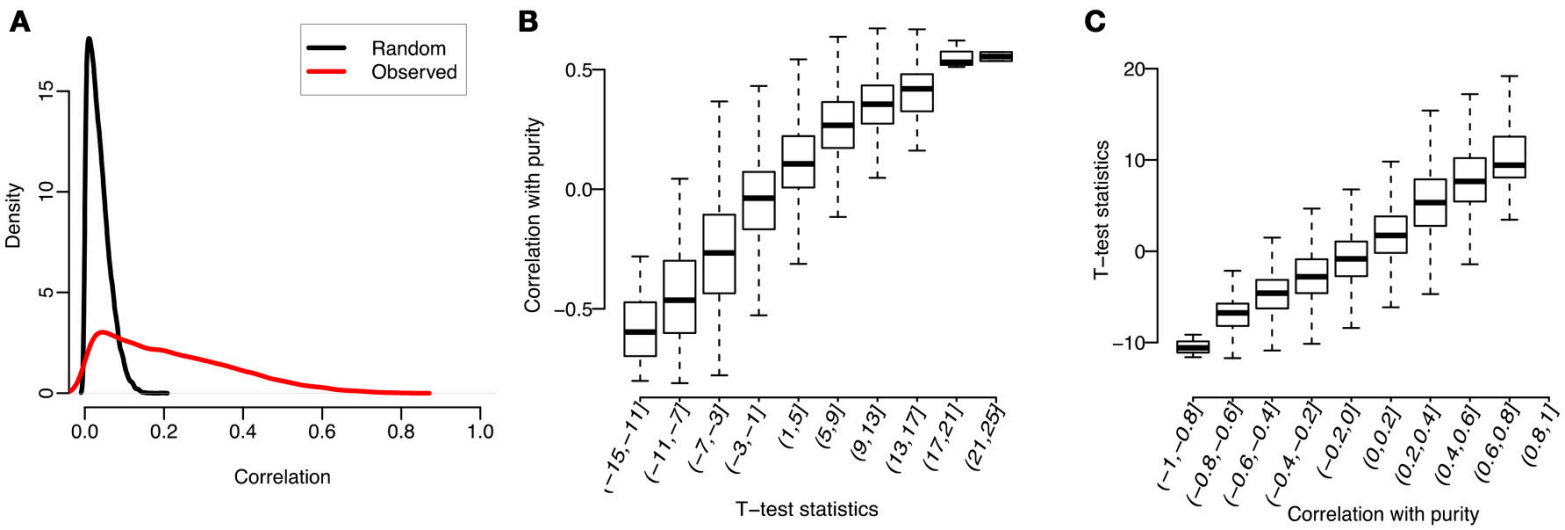
Correlations between tumor purities and gene expressions for PRAD: **(A)** Distributions of Spearman correlations between gene expression and observed or randomly shuffled tumor purities across tumor samples; **(B)** Observed correlations, grouped by *t*-test statistics; **(C)** *t*-test statistics, grouped by observed correlations.

We used Prostate adenocarcinoma (PRAD) in TCGA as an example to illustrate the correlation between gene expression and tumor purity. Specifically, after quantile-normalizing the expression profiles (quantified by RSEM, Li and Dewey, 2011) for tumor samples, the purity value of each sample was estimated by InfiniumPurify (Zheng et al., 2017). For each gene, we computed the Spearman correlation between expression levels and tumor purities across tumor samples (termed as “Observed” in Figure 1A). From there we obtained 20440 correlation values, each for a gene. As a comparison, we also randomly shuffled the purities of all tumor samples, and used the shuffled tumor purities as input to compute the correlation (termed as “Random” in Figure 1A). As can be seen from Figure 1A, the distribution of observed correlations has a longer right tail, demonstrating that there are much more genes with high correlation with tumor purity than by random. In particular, we identified 1252 genes with absolute observed correlation over 0.5 (accounting for 6.2% of all genes), while this number is close to 0 by random.

#### Correlation between gene expression and tumor purity increases with the difference of gene expressions between cancer and normal samples

We identified genes highly correlated with tumor purity. What are these genes? To answer this question, we studied the relationship between previously calculated correlations and gene expression changes between tumor and normal samples. Specifically, we first conducted a *t*-test on the normalized expression profiles of each gene between tumor and normal samples, and then divided all genes into 10 subsets by the test statistics. We then plotted in Figure 1B the distribution of observed correlations (between tumor purity and gene expression) in each group. As can be seen, the mean observed correlation in each group increases with the *t*-test statistics (measuring the extent of gene expression difference between tumor and normal samples). Similarly, we also classified the genes into 10 subgroups according to their correlations with tumor purity and observed a positive correlation between the *t*-test statistics and group labels (see Figure 1C).

We conducted the above analyses across 14 cancer types with sufficient normal tissues (each cancer type with over 10 normal samples) including Bladder Carcinoma (BLCA), Breast Invasive Carcinoma (BRCA), Esophageal Carcinoma (ESCA), Head-Neck Squamous Cell Carcinoma (HNSC), Kidney Chromophobe (KICH), Kidney Renal Clear Cell Carcinoma (KIRC), Cervical Kidney renal papillary cell carcinoma (KIRP), Liver Hepatocellular Carcinoma (LIHC), Lung Adenocarcinoma (LUAD), Lung Squamous Cell Carcinoma (LUSC), PRAD, Stomach Adenocarcinoma (STAD), Thyroid Cancer (THCA), and Uterine Corpus Endometrial Carcinoma (UCEC). The top 1000 genes with the largest correlations for each cancer type were shown in Supplementary Table S1. The results were similar for all cancers, which could be well explained by our linear regression model on gene expression (see Materials and Methods). When there are significant differences between tumor and normal samples (i.e., δ_*is*_ is big), the gene expressions are more correlated with purities. However, when there is no difference between tumor and normal samples (i.e., δ_*is*_ is close to 0), the gene expressions will have a low correlation with purities. These results revealed that tumor purity will bias differential expression analysis if not correctly accounted for, and our method was motivated from this observation.

### Analyses on simulated data

To evaluate DECtp and compare it with other methods, we simulated a few datasets resembling true biological scenarios with different sample sizes and noise levels.

#### Simulated datasets

We first downloaded from TCGA the LUAD gene expression data (in RSEM values) consisting of 517 tumor and 59 matched normal samples. Each RSEM value was transformed to log2 (RSEM + min), where min is the minimum non-zero RSEM value. The log2-transofmred data was quantile normalized, which was then used to generate simulation data.

It is worth mentioning that our purpose is to call DEGs between pure normal and pure tumor samples. However, both kinds of samples are infeasible to retrieve in reality, thus we adopted a compromised strategy as follows:

1. For each gene *i*, we simulated expression profile of “pure” normal sample *j* as $X_{ij}\sim N\left(m_{i},\sigma_{i}^{2}\right)$, where *m*_*i*_ is the mean expression of gene *i* across all 59 LUAD normal samples, and $\sigma_{i}^{2}\;$is their variance.
2. Similarly, we simulated expression profile of “pure” tumor sample *j* as $Y_{ij}\sim N\left(m_{i}^{{}^{\prime }},\sigma_{i}^{{}^{\prime }2}\right)$, where $m_{i}^{{}^{\prime }}\;$is mean expression across 517 LUAD tumor samples and $\sigma_{i}^{{}^{\prime }2}\;$is the variance. Since the two expression profiles (“pure” normal and “pure” tumor) are normally distributed, we assumed that gene *i* is a true DEG if $|m_{i}-m_{i}^{{}^{\prime }}|\geq \delta$, where δ is a predefined threshold.
3. We generated tumor purity values λ_*j*_ uniformly from [0.05, 0.95]. Plugging in *X*_*ij*_, *Y*_*ij*_ and λ_*j*_ into the formula $Y_{ij}^{{}^{\prime }}=\lambda_{j}Y_{ij}+\left(1-\lambda_{j}\right)X_{ij}$, we simulated $Y_{ij}^{{}^{\prime }}\;$as the observed expression profile of sample *j* at gene *i*, which is a mixture of expression profile from “pure” tumor and “pure” normal samples.

We then called DEGs between simulated pure normal (e.g., *X*_*ij*_) and mixed (e.g., $Y_{ij}^{{}^{\prime }}$) samples and compared them with the underlying true DEGs to assess accuracy. Because the true mean expression levels are known, we can construct a gold standard for comparison. For a gene, if the absolute difference of the true expression profiles between normal and pure tumor samples is greater than a threshold, it is defined as a DEG. The simulations were repeated for δ = 1, 2, 3, which roughly provides proportions of DEGs at 38%, 16%, and 8% of total number of genes. We also tested the performance of the algorithms with varied sample sizes from 10, 50, and 100, respectively.

#### DECtp outperforms other methods in simulated datasets

**Figure 2:**
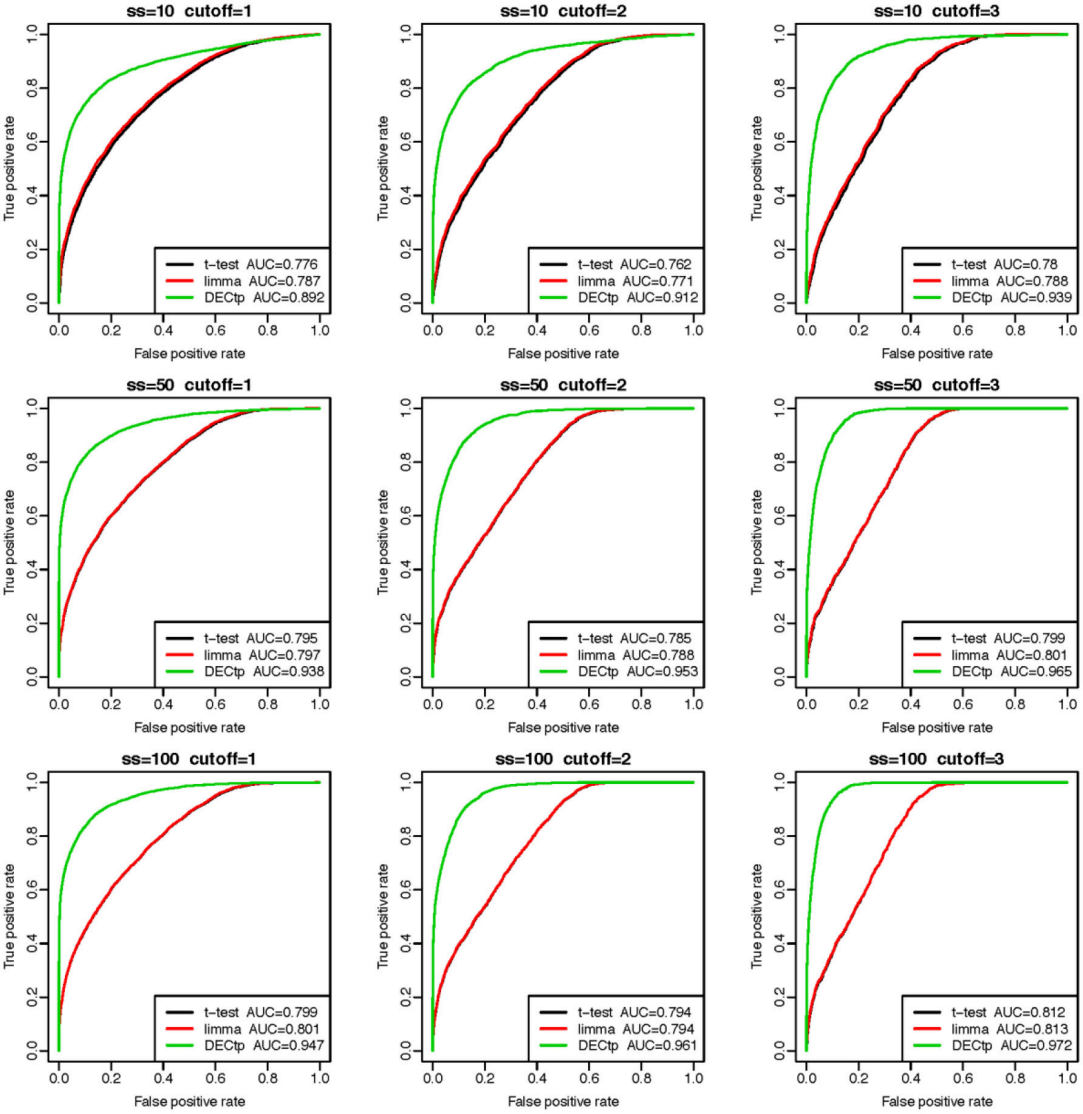
Comparison of DE detection accuracies of the three methods including *t*-test, limma and DECtp on 9 simulated datasets with sample sizes 10, 50, and 100 and cutoffs (δ) 1, 2, and 3.

We performed DEG calling on the 9 simulated datasets using DECtp and a few popular methods including *t*-test, limma. The receiver operating characteristic (ROC) curve analysis (Davis and Goadrich, 2006) using truth DEGs as a gold standard was performed to compare the performances of the methods (see Figure 2). Compared with traditional DEG calling methods, DECtp takes purity as an experimental design factor in a linear model. So we added to tumor purities a noise of the Gaussian distribution with mean 0 and standard deviations 0.1 to test the robustness of our method against purity estimation. It is clear that DECtp achieved the best AUCs in all simulated datasets even if estimated tumor purities are biased. In addition, limma and *t*-test have very similar performances, which is not surprising since it is known that they are similar for normal distributed data (Murie et al., 2009). Moreover, the performances of all methods became better when the thresholds (δ) or sample sizes increase as expected. Overall, these real data-based simulation results demonstrate the robustness and accuracy of DECtp in DE detection when tumor purity is a confounding factor.

### Analyses on real data

With the success of DECtp on simulated data, we next tested DECtp on real TCGA tumor data on 14 cancer types including BLCA, BRCA, ESCA, HNSC, KICH, KIRC, KIRP, LIHC, LUAD, LUSC, PRAD, STAD, THCA, and UCEC respectively. There are overall 6289 tumor and 632 normal samples. For all cancers, we estimated tumor purities by InfiniumPurify (Zheng et al., 2017).

#### The top differential genes identified by DECtp is more associated with tumor purity than those of limma

**Figure 3:**
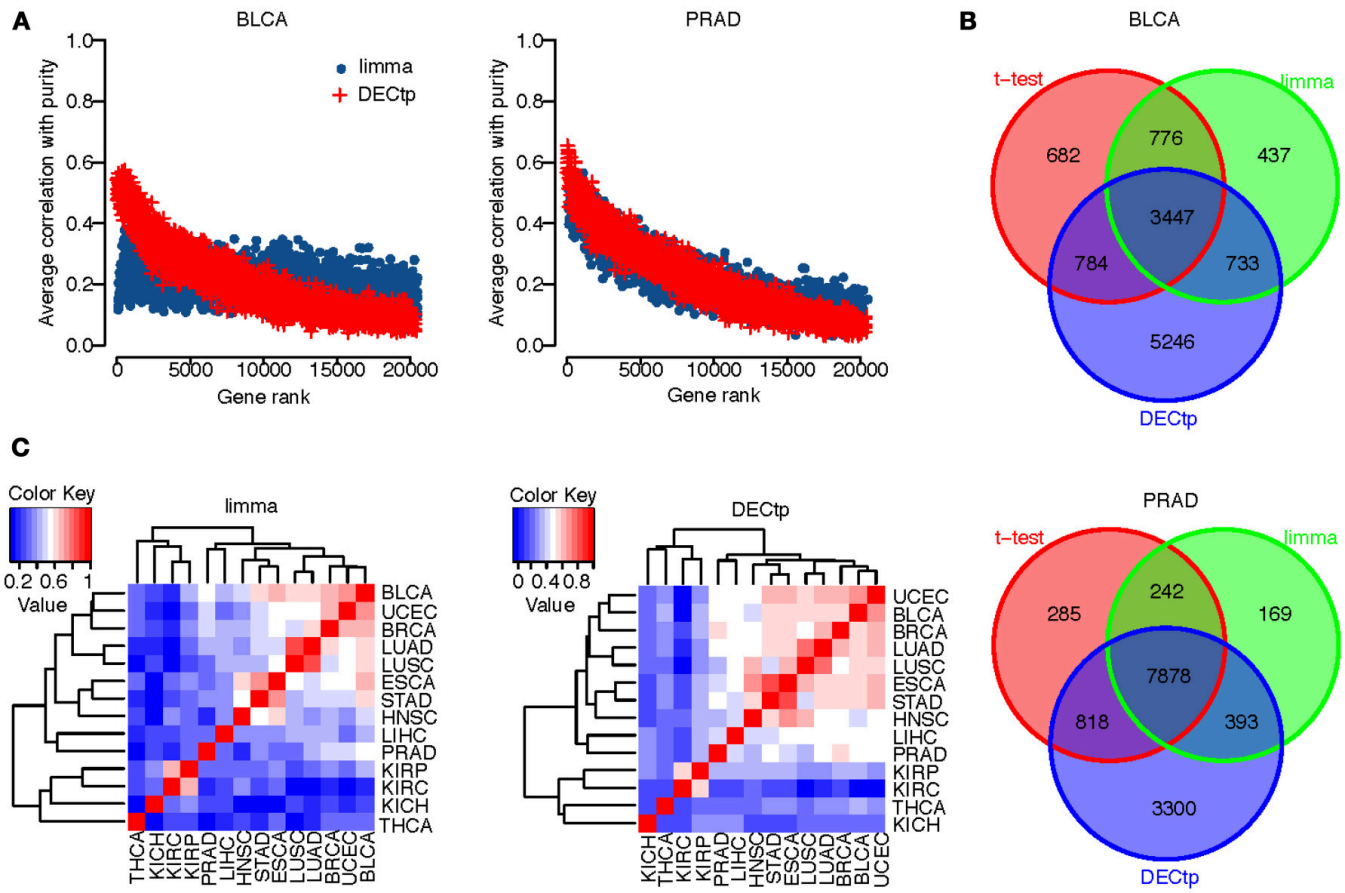
**(A)** Scatter plot of the number of top-ranked genes versus the average absolute correlations for BLCA and PRAD; **(B)** Overlaps of DEGs called from *t*-test, limma and DECtp for BLCA and PRAD; **(C)** Inter-cancer correlations of test statistics by limma and DECtp.

To study the correlation between tumor purity and top ranked differential genes, we first ranked genes by their false discovery rate calculated by DECtp or limma. We then calculated the average absolute correlation between tumor purity and top *n* ranked genes. In Figure 3A, we plotted the average absolute correlation against *n* (0 ≤ *n* ≤ 20000) for BLCA and PRAD. Similar to previous findings, we found that top differentially expressed genes are more correlated with purity than other genes for both DECtp and limma. The trend is clearer for DECtp, indicating that it is better in identifying tumor purity-associated differential genes. The observation holds for all 14 cancer types (see Supplementary Figure S1).

We also examined the overlaps of DEGs (at FDR 0.001) called from the *t*-test, limma and DECtp. Figure 3B shows the overlapping Venn diagrams for BLCA and PRAD respectively. For BLCA, the *t*-test identified 5,689 DEGs, among which 4,231 (74%) are overlapped with those identified by DECtp. limma identified 5,393 DEGs, among which 4180 (78%) are overlapped with those identified by DECtp. Similarly for PRAD, the *t*-test identified 9,223 DEGs, among which 8696 (94%) are overlapped with those identified by DECtp. Limma identified 8682 DEGs, among which 8271 (95%) are overlapped with DECtp. The overlaps of DEGs for other cancer types were shown in Supplementary Figure S2. In summary for all tested cancer types, there are 114842 DEGs overlapped between DECtp (with an overall of 151327 DEGs) and *t*-test (with an overall of 136918 DEGs), 107621 DEGs overlapped between DECtp and limma (with an overall of 121378 DEGs), 112772 DEGs overlapped between *t*-test and limma, suggesting that the three methods are generally consistent. We also have downloaded RNA-seq count data of six cancer types from TCGA, including BLCA, BRCA, HNSC, LUAD, LUSC, and PRAD to investigate the overlaps of DEGs called from DECtp, limma and edgeR. To have a fair comparison, we selected the same tumor and normal samples from the two different data type (count vs. RSEM value) when using DECtp and edgeR (332 normal samples versus 2858 tumor samples). The overlaps of DEGs for the three methods were shown in Supplementary Figure S3. It is shown that DEGs called from the three methods have rather significant overlap for the six cancer types. To be specific, for the six cancer types, limma identified 55593 DEGs, edgeR identified 59860 DEGs, and DECtp identified 71115 DEGs, and 44532 DEGs (accounting for 62.6%) in DECtp are overlapped with those identified by limma and edgeR.

Next, we examined the Pearson correlation among test statistics for different cancer types. Even though different cancer types have distinct etiologies, they might still share many genomic and transcriptomic features. We plotted in Figure 3C the correlation of test statistics among 14 cancer types using both DECtp and limma. Overall the correlations for DECtp are higher than those of limma.

#### DECtp identifies new biological meaningful differential genes

**Figure 4:**
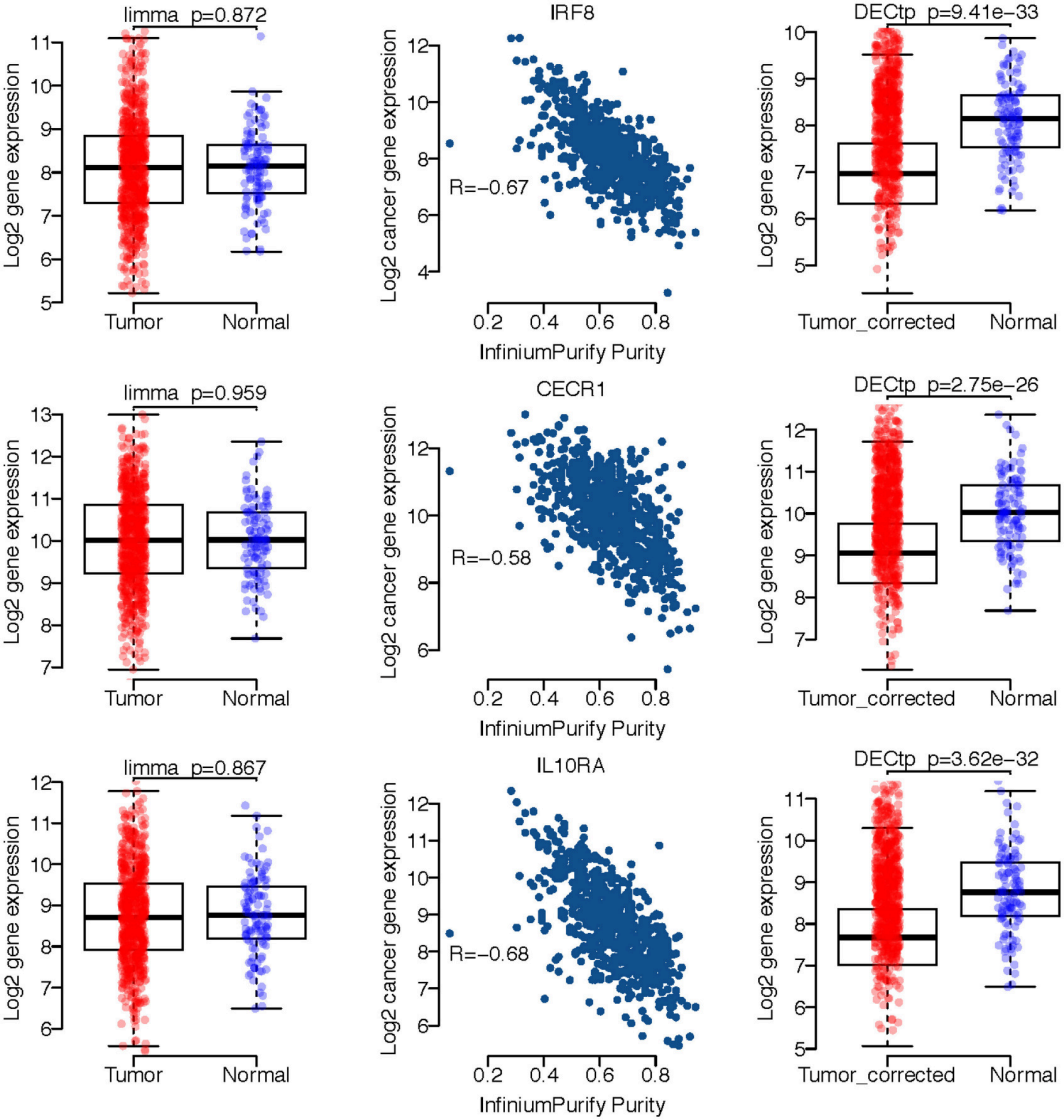
A few exemplified DEGs only detected by DECtp in BRCA. **Left** panel shows log2 expression profiles distributions for tumor and normal samples. **Middle** panel shows Spearman correlations between log2 expression profiles and tumor purities. **Right** panel shows log2 expression profiles for tumor and normal samples after adjusting tumor purities.

We selected several gene expression profiles from BRCA to demonstrate the confounding effect of tumor purity on differential expression analysis. As shown in Figure 4, the left panel displays the boxplots of three genes expression profiles including *IRF8, CECR1* and *IL10RA* for tumor and normal samples. It is clear that the *p*-values are not statistically significant for limma, i.e., the *p*-value is 0.872 for *IRF8*, 0.959 for *CECR1*, and 0.867 for *IL10RA*. The middle panel shows the scatter plot of expression profiles versus InfiniumPurify purities, in which the correlations are all very high, especially, −0.68 for *IL10RA*. The high correlation indicates that the large within group variance of cancer samples is mostly caused by variation in purities for different samples, which dilutes the signals of DEGs. And thus, after removing the effect of tumor purity, we could observe significant difference on expressions of these genes between normal and tumor groups. Indeed, there are many studies linking these 3 genes to breast cancer (Heinonen et al., 2008; Takaoka et al., 2008; Pavlides et al., 2010). We also selected the differentially expressed genes detected only by DECtp for the David enrichment analysis (at FDR < 0.05). Supplementary Table S2 shows the enrichment of DE genes for the 10 cancer types. We have obtained a lot of biological functions. For example, GO:0006955~immune response is the most enriched Go term for BLCA and PRAD with FDR being 6.161542e-29 and 1.45e-12, respectively. Thus, by considering tumor purity, DECtp could identify new biological meaningful DEGs for further experimental validation.

#### DECtp is more consistent and identifies more biological meaningful differential genes than limma

**Figure 5:**
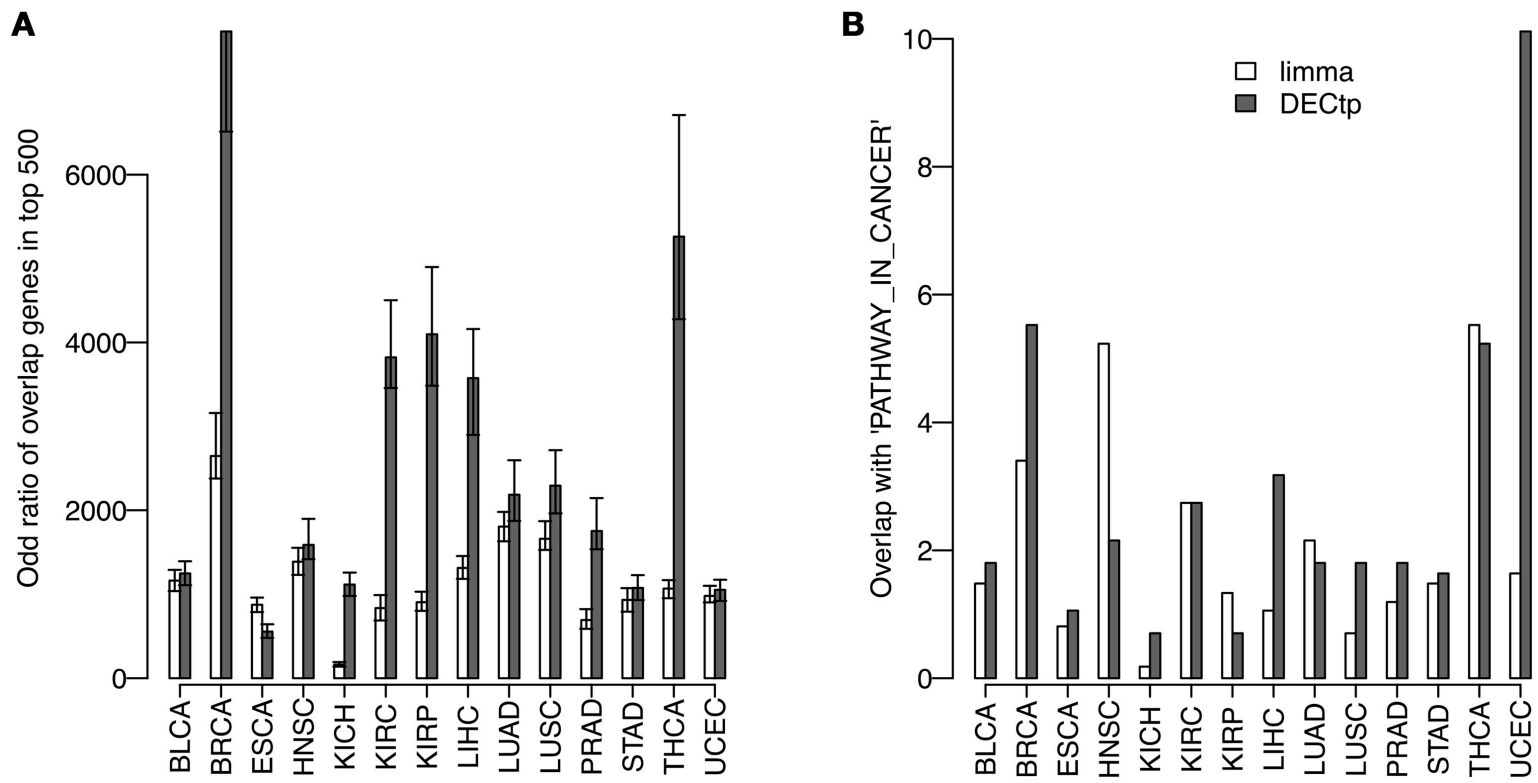
Comparing the DEGs called by limma and DECtp: **(A)** The overlapping odd ratios of the top 500 DEGs between biological replicates for all 14 cancers; **(B)** Enrichment *p*-values for top 4,000 differentially expressed genes within “PATHWAY-IN-CANCER” from KEGG.

It is known to us all that consistency is a very important criteria to evaluate DE calling methods on real data. Generally speaking, a robust method should obtain consistent results on technical or biological replicates. To compare the consistency of DECtp with that of limma, we randomly divided tumor samples in each cancer into two groups, and then detected DEGs by comparing the two tumor groups with normal samples, respectively. This process was repeated 50 times. Figure 5A shows the overlapping odd ratios of the top 500 DE genes for all 14 cancers. Clearly, DECtp detected more overlapped DE genes than those of limma in most cancer types, which suggests that it is more consistent. We then examined the biological implications of the DE calling results. To have a fair comparison, we selected top 4,000 differential genes by the two methods, and tested their enrichments with “PATHWAYS_IN_CANCER” from KEGG (Kanehisa and Goto, 2000), which contains 328 biologically meaningful genes. DECtp detects 110 genes compared to 80 genes by limma in UCEC. Figure 5B shows the –log10 of the *p*-values for the enrichment of DEGs in “PATHWAYS_IN_CANCER” by using the Chi-square test. As can be seen, DECtp shows much smaller *p*-values compared to limma in most cancer types, especially in UCEC and BRCA. Overall, these results suggest that DECtp can detect more enriched DEGs in “PATHWAYS_IN_CANCER” than limma.

## Discussions

In this work, we systematically investigated the impact of tumor purity as a confounding factor in differential expression analysis (Aran et al., 2015; Wang et al., 2015; Shen et al., 2016), and proposed a novel statistical model to adjust for tumor purity in DE calling. We first examined the correlations between cancer expression profiles and tumor purity, and found that DE genes have high correlations with tumor purity. It is known that tumor purity has multiplicative effect on gene expression, instead of additive, so traditional DE calling methods ignoring tumor purity or modeling it as an additive covariate may present biased results. To solve this problem, we proposed DECtp, in which gene expression profiles from tumor samples are modeled as mixed Gaussian distributions, where the mixing proportion is tumor purity. DECtp achieved more robust and accurate DEGs in both simulation and real data studies compared with canonical methods like limma, which reinforces our previous claim that tumor purity may confound genomic analyses if not correctly accounted for (Zhang et al., 2017; Zheng et al., 2017).

DECtp is specifically developed to identify DEGs for gene expression profiles admitting normal distributions. However, RNA-sequencing technology has led to a rapid increase in gene expression data in the form of counts. The counts data are usually modeled by the negative binominal (NB) models, thus DECtp cannot be directly applied. In the future, it will be interesting to develop similar models using the NB distributions incorporating tumor purity information.

Finally, we would like to point out that DECtp may have a few further applications. Similar to differential gene analysis, differential protein and differential methylation analyses have also been widely performed between cancer and normal samples. In principle, DECtp could be applied to any differential analysis between cancer and normal samples given the data is Gaussian. In addition, Aran et al. found that identifying co-expression networks from genomics data without accounting for tumor purity is problematic (Aran et al., 2015). So we believe that similar principals proposed in this work can be applied to analyzing gene co-expression. Moreover, tumor purity information might be useful in identifying cancer associated expression quantitative trait loci (eQTLs). However, it is out of the scope of this study.

## Author contributions

WZ and JY conceived the concept of the work. WZ, BH, and HL performed the experiments. WZ, JY, and BH wrote the paper.

### Conflict of interest statement

The authors declare that the research was conducted in the absence of any commercial or financial relationships that could be construed as a potential conflict of interest.
