# Supplementary material for: DECtp: Calling Differential Gene Expression Between Cancer and Normal Samples by Integrating Tumor Purity Information
Source: Front Genet. 2018 Aug 28;9:321. doi: 10.3389/fgene.2018.00321 (PMC6121016; doi:10.3389/fgene.2018.00321)

Supplementary information for DECtp: calling differential gene expression between cancer and normal samples by integrating tumor purity information

Weiwei Zhang^1^, Haixia Long^2^, Binsheng He^3,*^, Jialiang Yang^3,4,*^

1 East China University of Technology, Nanchang, Jiangxi 330013, China; wwzhangly@163.com (W.Z.)

2 Department of Information Science and Technology, Hainan Normal University, Haikou, Hainan 571158, China; [myresearch_hainnu@163.com](mailto:myresearch_hainnu@163.com) (H.L.)

3 College of Information Engineering, Changsha Medical University, Changsha, Hunan 410219, China

4 Icahn Institute for Genomics and Multiscale Biology, Icahn School of Medicine at Mount Sinai, New York, NY 10029, USA

**Figure S1.** Scatter plot of the top-ranked genes versus the average absolute correlation of each 10 genes for TCGA 12 cancer types.


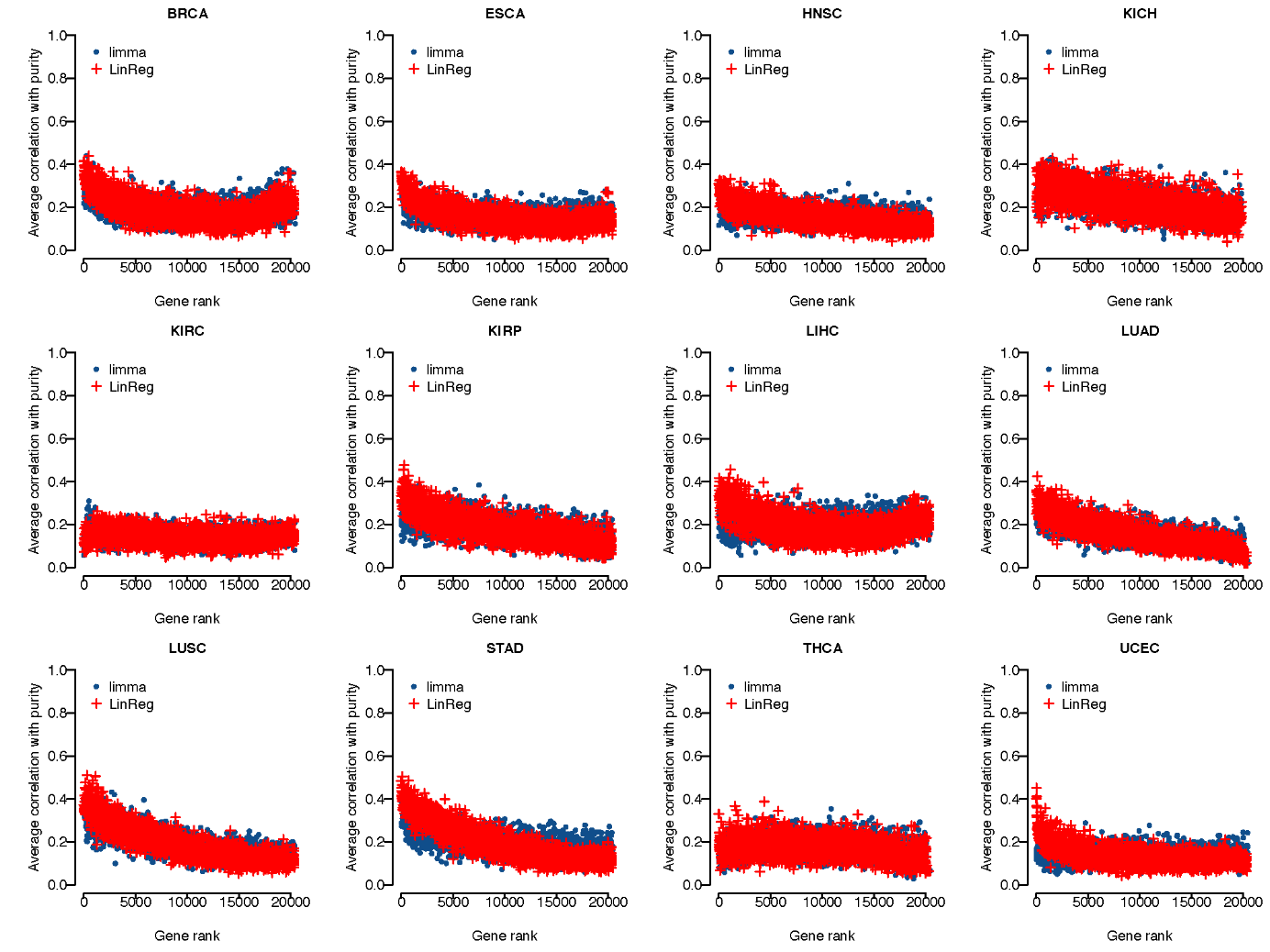


**Figure S2.** Overlaps of DE genes called from t-test, limma and DECtp for TCGA 12 cancer types.


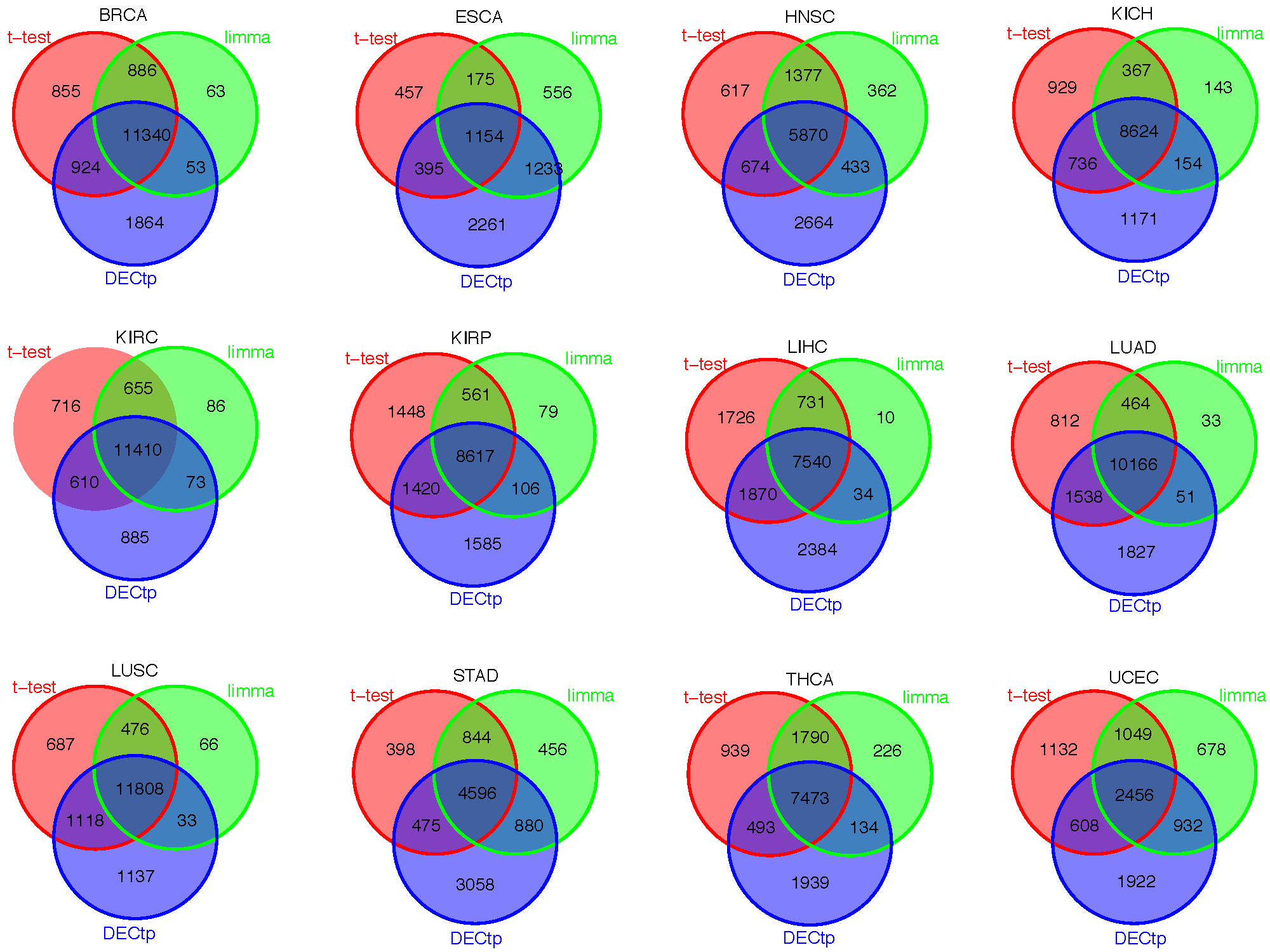


**Figure S3.** Overlaps of DE genes called from limma, edgeR and DECtp for TCGA 6 cancer types.


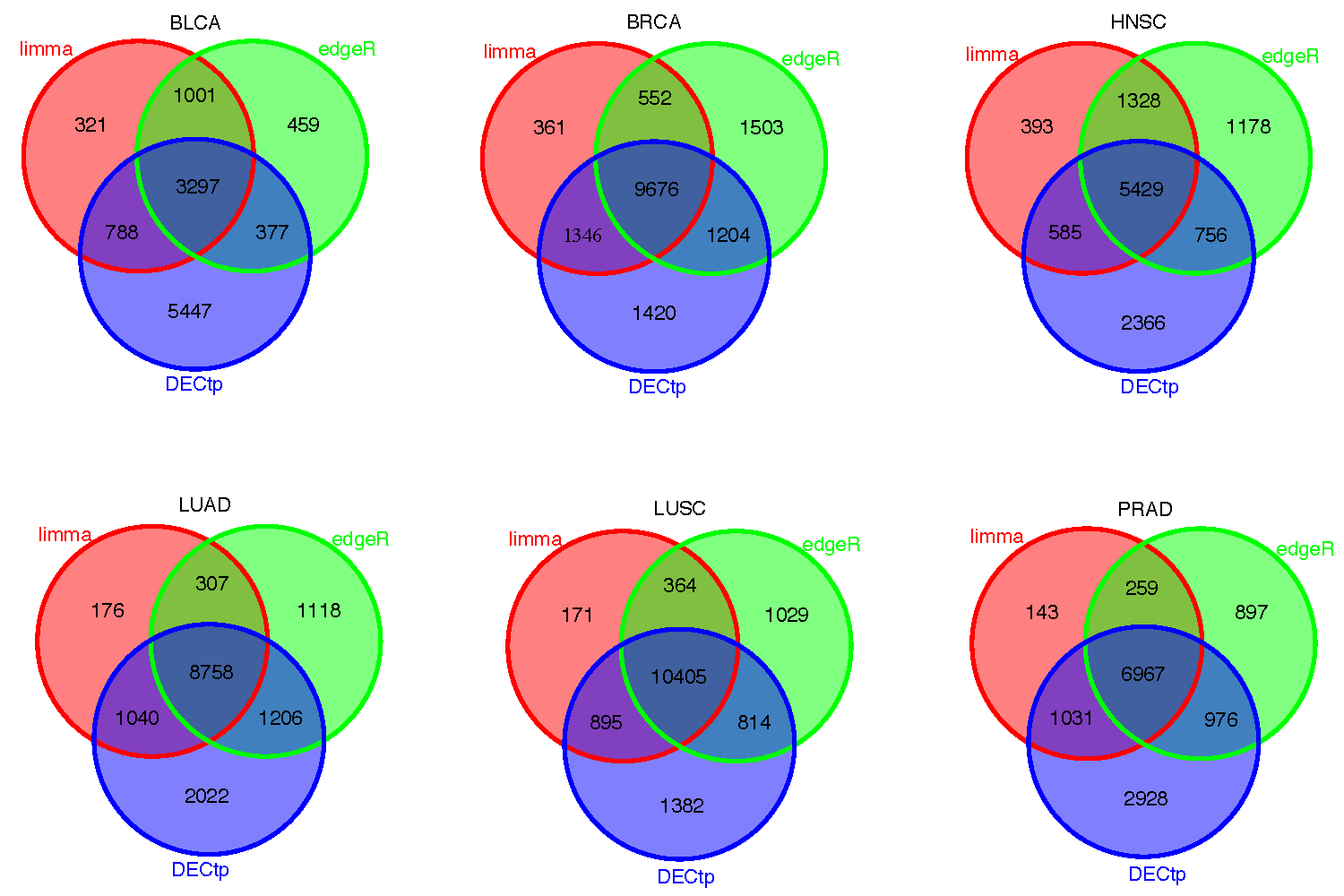

Supplement: Supplementary file 1 [file Data_Sheet_1.docx]
